# Identification and Clinical Analysis of the First Nonsense Mutation in the *PSEN1* Gene in a Family With Acute Encephalopathy and Retinitis Pigmentosa

**DOI:** 10.3389/fneur.2020.00319

**Published:** 2020-05-05

**Authors:** Chunlin You, Weike Zeng, Lingna Deng, Zhihao Lei, Xinyi Gao, Victor Wei Zhang, Yidong Wang

**Affiliations:** ^1^Department of Neurology, Sun Yat-sen Memorial Hospital, Sun Yat-sen University, Guangzhou, China; ^2^Department of Neurology, The University of Hong Kong-Shenzhen Hospital, Shenzhen, China; ^3^Department of Radiology, Sun Yat-sen Memorial Hospital, Sun Yat-sen University, Guangzhou, China; ^4^Scientific Research Center and Department of Orthopedic, The Seventh Affiliated Hospital, Sun Yat-sen University, Shenzhen, China; ^5^Department of Neurology, Shenzhen Second People's Hospital, Shenzhen, China; ^6^AmCare Genomics Laboratory, GuangZhou, China; ^7^Department of Molecular and Human Genetics, Baylor College of Medicine, Houston, TX, United States; ^8^Guangdong Province Key Laboratory of Brain Function and Disease, Zhongshan School of Medicine, Sun Yat-sen University, Guangzhou, China; ^9^Guangdong Provincial Key Laboratory of Malignant Tumor Epigenetics and Gene Regulation, Sun Yat-sen Memorial Hospital, Sun Yat-sen University, Guangzhou, China

**Keywords:** acute encephalopathy, retinitis pigmentosa, nonsense mutation, *PSEN1* gene, vision impairment

## Abstract

In the present study, we investigated the genetic variation in a family with acute encephalopathy and retinitis pigmentosa. **Nine** of 25 people in this family underwent genetic testing. Three family members, namely, the proband and the proband's two sisters, showed symptoms resembling those of meningoencephalitis and simultaneously suffered from retinitis pigmentosa. Whole-exome sequencing and Sanger sequencing identified a heterozygous mutation, chr14: 73673106 c.881G>A (p.W294^*^), in the presenilin 1 (*PSEN1*) gene in these three family members, and the SWISS-MODEL server predicted the formation of a truncated protein. This mutation was not found in the asymptomatic family members. This mutation is a newly discovered nonsense mutation that results in a truncated protein. Although the current genetic evidences may indicate the likelihood of association, further investigations are needed to establish the genotype and phenotype relationship.

## Introduction

The presenilin 1 (*PSEN1*) gene is located on chromosome 14q24.2 (1), and the encoded presenilin 1 (PS1) protein is widely present in all tissues. As subunits, presenilin 1 or presenilin 2, together with presenilin enhancer 2 (Pen-2), nicastrin, and anterior pharynx-defective 1 (Aph-1), constitute γ-secretase, which is involved in the cleavage and hydrolysis of the β-amyloid precursor (2). *PSEN1* mutation results in an increase in β-amyloid (mainly Aβ42), which is currently thought to be an important mechanism of the pathogenesis of Alzheimer's disease (3, 4). PS1 also controls the Notch signaling pathway and mediates other physiological activities, such as regulation of the Wnt/beta-catenin signaling pathway, modulation of phosphatidylinositol 3-kinase/Akt and MEK/ERK signaling, trafficking of select membrane proteins and/or intracellular vesicles, trafficking and turnover of epidermal growth factor receptor (5), modulation of phospholipase C and protein kinase C signaling (6), and regulation of calcium homeostasis (7, 8). Animal studies of *PSEN1* gene knockout have shown that loss of function can lead to neuronal generation disorders or degenerations, heart failure, longitudinal bone formation disorders (9–11), et al.

Currently, as many as 309 *PSEN1* mutations have been described (http://www.alzforum.org/mutations/search? genes[]=493). Most of these mutations are missense mutations, and the others are deletion/insertion mutations, but are all in-frame mutations. These mutations are the most common cause of early-onset Alzheimer's disease accompanied by seizures, spastic paraparesis, myoclonus, and pyramidal, extrapyramidal, or cerebellar signs. Some *PSEN1* mutations have also been reported in patients with other diseases, including frontotemporal dementia, dementia with Lewy bodies, Pick's disease, cerebral amyloid angiopathy, subcortical dementia, amyotrophic lateral sclerosis, progressive non-fluent aphasia, dilated cardiomyopathy, and familial acne inversa (http://www.alzforum.org/mutations/search? genes[]=493). In this study, using whole-exome sequencing, we discovered the first nonsense mutation in the *PSEN1* gene in a family whose members presented with acute encephalopathy as the prominent symptom and simultaneously suffered from retinitis pigmentosa. We also analyzed the clinical features of these family members.

## Materials and Methods

### Clinical Findings

The proband (III:6), a 20-year-old male, was hospitalized at the Department of Neurology, Sun Yat-sen Memorial Hospital, Sun Yat-sen University, China, in June 2016 due to a fever and unconsciousness. The patient had a history of decreased visual acuity before symptom onset. According to the family history, the patient's two sisters (III:4, III:5) had been hospitalized for similar symptoms; the patient's deceased mother (II:2) had been hospitalized for decreased visual acuity, headache, and slow responses; and the patient's deceased maternal grandfather (I:1) had a history of decreased visual acuity and paroxysmal confusion. Based on this family history, we suspected the existence of hereditary disease in this family. Neurological physical examination results and detailed family history were collected from the proband, and a pedigree chart of this family was generated. The inpatient medical records of the two sisters and mother of the proband were also reviewed. In November 2016, the proband and his two sisters returned to the clinic for a follow-up visit and completed auxiliary examinations. This study was approved by the Ethics Committee of Sun Yat-sen Memorial Hospital, Sun Yat-sen University, China. All family members who underwent genetic testing signed an informed consent form.

### DNA Purification and Genetic Testing

To identify the causal genetic variant, we collected peripheral venous blood samples from the proband and his asymptomatic father. Genomic DNA was extracted using a salting out method, and whole-exome sequencing technology was used to identify suspected mutation sites responsible for the disease. Briefly, genomic DNA was extracted using the SolPure Blood DNA kit (Magen) according to the manufacturer's instructions. Paired-end libraries were prepared following the Illumina library preparation protocol. Enriched DNA samples were indexed and sequenced on an Illumina sequencer (Illumina, San Diego, CA) with 150 cycles of pair-end reads. Primary data were collected in Fastq format after image analysis, and base calling was conducted using the Illumina Pipeline. Sequencing reads were mapped to the reference human genome version hg19 (2009-02 release, http://genome.ucsc.edu/).

To verify and confirm the source of the gene mutation in the family, the genomic DNAs of the proband, his sisters, his father, his maternal aunts and uncles, and his maternal grandmother were simultaneously sequenced *via* Sanger sequencing. All genomic DNA samples were extracted using a salting out method. Verification of the mutation was then performed. First, the nucleotide sequence of the gene fragment containing the mutation site was obtained from the UCSC website (http://genome-asia.ucsc.edu/). A primer for this gene fragment was designed using Primer 3.0. Then, the corresponding target gene fragment was amplified from the genomic DNA samples using polymerase chain reaction (PCR). Finally, the amplified DNA fragments were verified using Sanger sequencing. Nucleotide changes in aligned reads were called and reviewed using NextGENe software (SoftGenetics, State College, PA). Sequence variants were annotated using population and literature databases including 1,000 Genomes, dbSNP, gnomAD, ClinVar, HGMD, and OMIM.

### Protein Structure Prediction

The SWISS-MODEL online server was used to predict whether the novel mutation site was pathogenic and/or changed the structure of the resulting protein. The amino acid sequences of wild-type and mutant genes were submitted to the SWISS-MODEL website (https://swissmodel.expasy.org/interactive), and a PDB file predicting the protein structure was generated by the online server. Then, the PDB file was opened using Swiss-Pdb Viewer software, and a three-dimensional (3D) structural model of the predicted protein was generated after adjusting the image color and angle.

## Results

### Clinical Data

**Figure 1:**
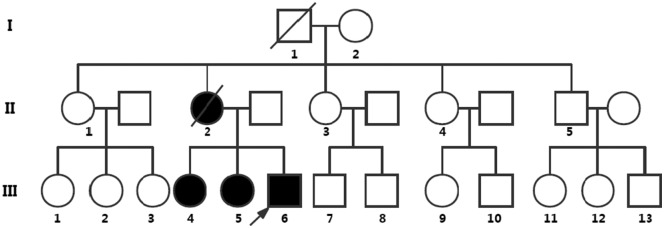
Pedigree chart representing the family history of the three patients. Symbols: the roman numbers to the left of the pedigree denote generations; the arrow denotes the proband of the pedigree; the circles indicate females, and the squares indicate males; fully black symbols denote the affected individuals evaluated; the diagonals indicate death before genetic testing. Nine of 25 people in this family underwent genetic testing. III:4, III:5, and III:6 show symptoms resembling meningoencephalitis and simultaneously suffer from retinitis pigmentosa and have the nonsense mutation of *PSEN1* [c.881G>A (p.W294^*^)], which is not found in the asymptomatic family members (the proband's father, I:2, II:1, II:3, II:4, and II:5). II:2 manifested symptoms of meningoencephalitis and vision loss, but died before genetic testing. Other members did not perform the genetic testing.

The proband's family has a total of 25 members comprising three generations (Figure 1). The clinical manifestations of the proband and his two sisters were similar. The proband's mother and maternal grandfather also had a similar medical history but were deceased. Other family members did not show any similar symptoms.

**Figure 2:**
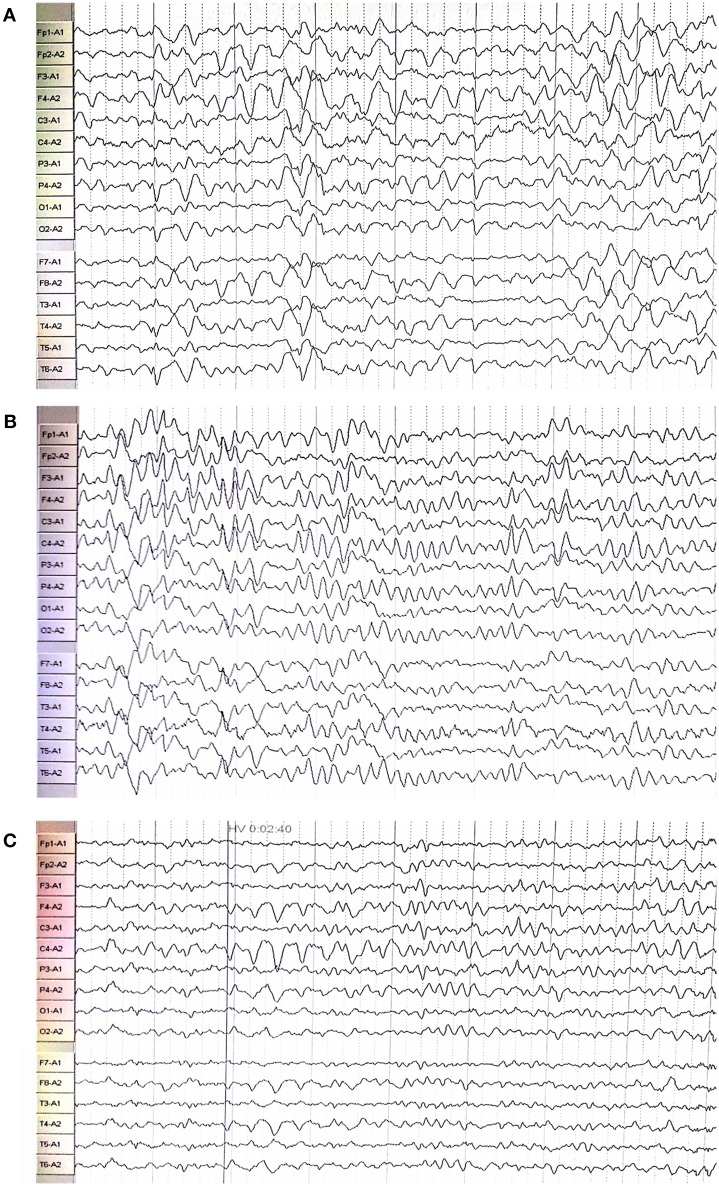
Electroencephalography showed extensive abnormal waves in the proband **(C)**, his oldest sister **(A)**, and second oldest sister **(B)**.

**Figure 3:**
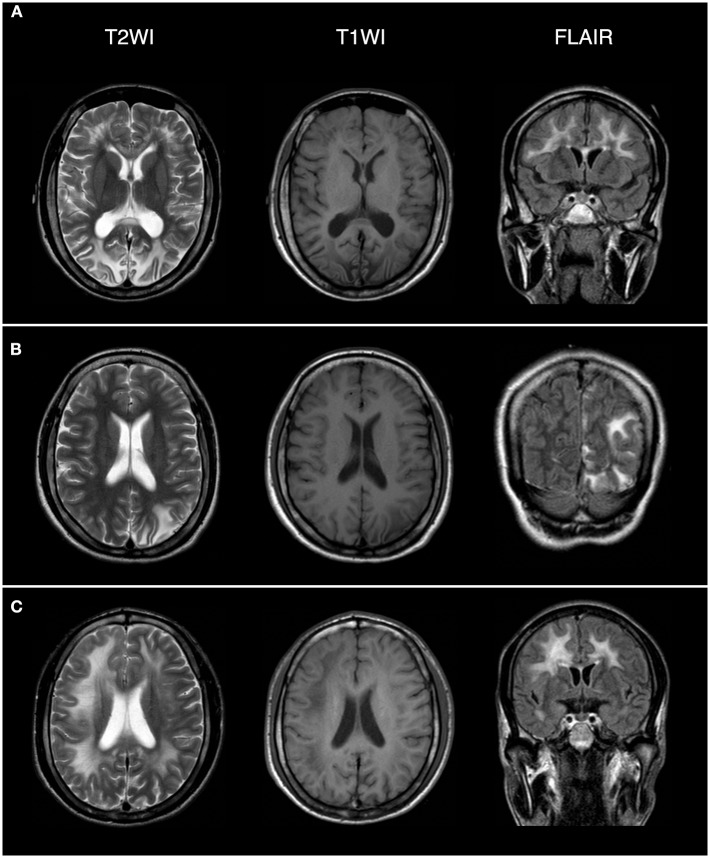
Brain magnetic resonance imaging (MRI) revealed extensive lesions in the white matter and subcortex in the proband **(C)**, his oldest sister (**A**), and second oldest sister (**B**). The field intensity of MRI is 1.5T. The left side of the picture corresponds to the right side of the human body.

**Figure 4:**
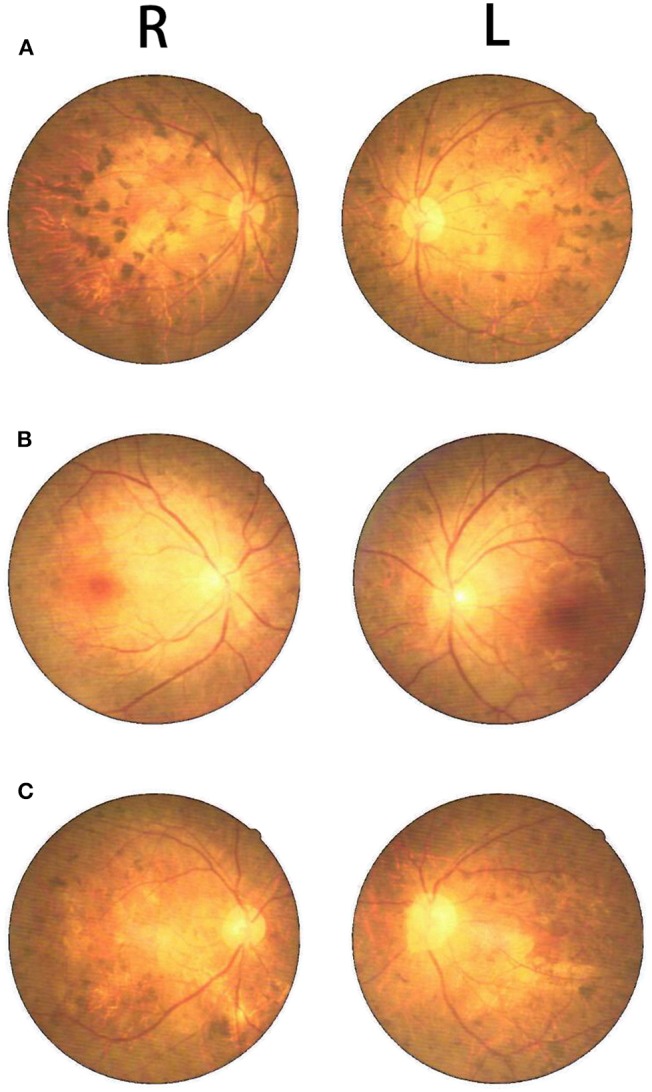
Fundus photography showed retinal pigmentation in the proband **(C)**, his oldest sister **(A)**, and second oldest sister **(B)**.

The proband (III:6, male) had experienced a progressive decline in bilateral vision and blurred vision since the age of 13. At the age of 20, in June 2016, he was admitted to the hospital with primary complaints of “fever, headache, vomiting, and unconsciousness for 1 day.” The results of the physical examination showed subcoma, clasp-knife spasticity of the upper and lower extremities, visible voluntary movement in the right extremities, no movement of the left extremities, increased tendon reflexes in all four limbs, positive bilateral Babinski reflex and Gordon's sign, chin to chest distance of approximately four transverse fingers, and positive meningeal irritation signs. The primary diagnosis was “suspected viral meningoencephalitis.” The peripheral white blood cell (WBC) count was markedly high, but the cerebrospinal fluid (CSF) results showed no evidence of infection, although an elevated protein level was noted (Table 1). After admission, the patient's condition worsened, and he presented with convulsions, severe pulmonary infection, and upper gastrointestinal bleeding despite acyclovir (0.5 g, three times a day), cefoperazone/sulbactam (3.0 g, two times a day), and clindamycin (0.6 g, two times a day) injections. Ten milligrams of dexamethasone and 60 mg of methylprednisolone were injected one time on the first 2 days of admission, respectively. After 7 days of hospitalization, the patient was discharged and was admitted to another hospital for treatment. Later, the patient's symptoms improved. He regained consciousness and limb function, but his visual acuity rapidly decreased. When the patient returned to the clinic for a follow-up visit in November 2016, he was conscious, and his visual acuity was at the level of counting fingers at 1 meter for the right eye and at 20 cm for the left eye. The muscle strength of all extremities was 5/5, and his Mini-Mental State Examination (MMSE) score was 28 points. Electroencephalography (EEG) showed extensive abnormal δ waves (Figure 2C). Magnetic resonance imaging (MRI) of the brain revealed extensive lesions in the white matter and subcortex in the bilateral hemispheres (Figure 3C). Fundus photography showed moderate retinal pigmentation in his eyes (Figure 4C).

**Table 1:** Peripheral blood and cerebrospinal fluid results of patients II:2, III:4, III:5, and III:6.

**Patient number**	**Peripheral blood**		**Cerebrospinal fluid**								
	**White blood cells (10^**9**^/L)**	**Neutrophil percentage (%)**	**pressure (mmH_**2**_O)**	**White blood cells (×10^**6**^/L)**	**Red blood cells (×10^**6**^/L)**	**Protein (g/L)**	**Chloride (mmol/L)**	**Glucose (mmol/L)**	**Acid-fast bacilli**	**Bacterial culture**	***Cryptococcus neoformans*/fungal culture**
II:2	12.31	58.5	55	0	0	1.29	119.3	3.5	–	–	–
III:4	10.30	66.2	102	6	10	0.9	121.4	3.1	–	–	–
III:5	14.87	83.5	125	0	2	3.66	120.4	3.3	–	–	–
III:6	18.30	70.9	100	13	82	1.12	119.6	4.2	–	–	–

The proband's oldest sister (III:4) had experienced ~30-min episodes of paroxysmal left-sided paresthesia since the age of 13. At the age of 17, in March 2008, she was admitted to the hospital with the primary complaint of “fever with unconsciousness for 2 days.” The physical examination results were as follows: lethargy, nuchal rigidity in the right extremities, low muscle tone (muscle strength 0–1/5), decreased tendon reflexes, negative pathological reflex, and left extremity visible voluntary movement with a positive Babinski reflex. She was diagnosed with “suspected viral meningoencephalitis.” Her peripheral WBC count was slightly increased, but the CSF results showed no evidence of infection, except for a mildly elevated protein level (Table 1). After treatment with acyclovir (0.5 g, three times a day for 2 weeks), penicillin (3.2 MU, three times a day for 7 days), ceftriaxone (2.0 g, two times a day for 7 days), and methylprednisolone (started with 80 mg/day and gradually stopped within 2 months) initiated on the second day of admission, she regained consciousness and limb function. However, the visual acuity of both eyes progressively decreased, her reactions became slower, and she also experienced paroxysmal left-sided paresthesia, lasting ~20–30 min. When she was examined at a follow-up appointment in November 2016, she had complete vision loss, a left upper extremity muscle strength of 5^−^/5, left-sided decreased pain sensation, and an MMSE score of 26 points. EEG showed an extensive, sharp slow-wave complex (Figure 2A). Brain MRI revealed extensive lesions in the white matter and subcortex in the bilateral hemispheres (Figure 3A). Fundus photographs showed serious retinal pigmentation in both eyes (Figure 4A).

In 2013, the proband's second oldest sister (III:5) was admitted to the hospital at the age of 19, with primary complaints of “vomiting, mental abnormality for 2 days, and a fever for 1 day.” The physical examination results were as follows: alert, irritable, not cooperative with muscle strength and muscle tone examinations, positive right Babinski reflex, chin to chest distance of approximately four transverse fingers, and positive meningeal irritation signs. The initial diagnosis was “suspected viral meningoencephalitis.” Her peripheral WBC count was high, but her CSF did not suggest inflammation, except for a markedly increased protein level (Table 1). After treatment with acyclovir (0.5 g, three times a day for 1 day), ceftriaxone (2 g/day for 7days), and methylprednisolone (started with 80 mg/day and gradually stopped within 1 month) initiated on the day of admission, her psychological symptoms disappeared, and extremity movement returned to normal. However, she presented with memory loss, slow responses, poor night vision, paroxysmal bilateral blurry vision, tongue numbness, slurred speech, and numbness and weakness in the right upper and lower extremities, lasting ~10 min. At the follow-up period in November 2016, her right eye vision was 4.9, her left eye vision was 5.0, the muscle strength of all extremities was 5/5, and her MMSE score was 29 points. Her EEG showed extensive abnormal θ waves (Figure 2B). Brain MRI revealed some lesions in the left occipital and parietal lobes (Figure 3B). Fundus photographs showed mild retinal pigmentation in both eyes (Figure 4B).

The proband's mother (II:2) began to show a progressive decline in bilateral vision at the age of 24. Since the age of 28, she had experienced repeated headaches with numbness of the limbs, fatigue, and slow reactions. She was hospitalized due to exacerbation of the above symptoms at the age of 38 in March 2007. The physical examination results were as follows: left eye vision loss, right eye light perception, decreased sensation in the left face and extremities, increased muscle tone in the upper extremities, muscle tone intact in the lower extremities, muscle strength of all extremities 4/5, positive bilateral Babinski reflex, and a positive meningeal irritation sign. During hospitalization, she exhibited two ~1-min episodes of a deviation of both eyes and the head to the left, which was accompanied with a muscle spasm in the left upper extremity. She had no fever, but her peripheral WBC count was high (Table 1). The CSF was not anormal except for an increased protein level (Table 1). She was discharged after 5 days due to limited financial resources and died at 40 years of age from an unknown cause.

The proband's maternal grandfather (I:1) started to exhibit decreasing bilateral vision at ~30 years old. Starting at ~50 years old, he presented with episodic confusion lasting ~1–3 days. When he was sober, he could care for himself. He died at the age of 58 from unknown reasons.

### Mutation Analysis

**Figure 5:**
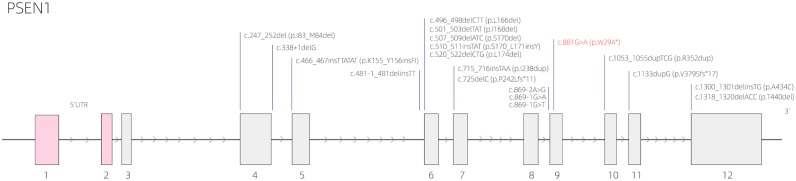
The presenilin 1 (*PSEN1*) gene structure with the nonsense mutation [c.881G>A (p.W294^*^)] as well as other deletion/insertion mutations.

The results of whole-exome sequencing demonstrated that the proband (III:6) harbored a heterozygous mutation in *PSEN1* gene: NM_000021.4:c.881G>A (p.W294^*^); HG19: chr14: 73673106 (hereinafter referred to as “chr14: 73673106 c.881G>A”), which was a nonsense mutation. The Combined Annotation Dependent Depletion (CADD) score is 40, and CADD prediction is Damaging. No corresponding genetic mutation was found in his father's genome. This mutation site is located in the *PSEN1* coding region, which is important for encoding the presenilin 1 protein. The *PSEN1* gene structure with this nonsense mutation as well as other deletion/insertion mutations is indicated in Figure 5. The known gene variants of hereditary leukoencephalopathy (such as SUMF1, GFAP, PLP1, ABCD1, NOTCH3, HTRA1) and retinitis pigmentosa (https://sph.uth.edu/Retnet/) were not found.

**Figure 6:**
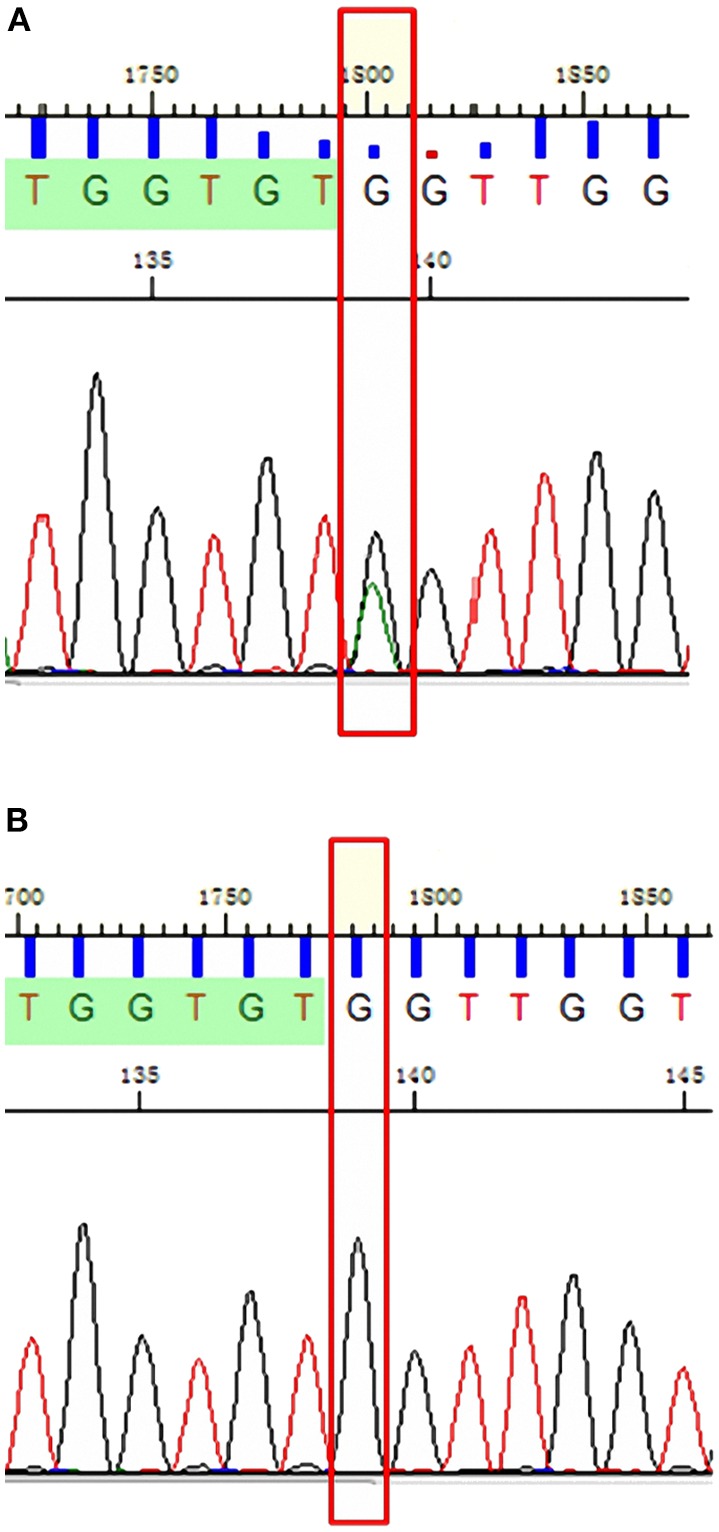
Sanger sequencing confirmed chr14: 73673106 c.881G>A heterozygous mutations in the proband and his two sisters **(A)** but not in his father, maternal aunts (II:1, II:3, and II:4) and uncle (II:5), and maternal grandmother (I:2) **(B)**.

The primers used for Sanger sequencing were PSEN1-chr14:73673106:F -TGCTAAAACCAAAGAGAACCTTTT and PSEN1-chr14:73673106:R-CAGTGACCCTGAAAAATCAAG. The results showed that this mutation was also present in the two sisters of the proband (III:4, III:5), but the mutation was not detected in the other six family members, including I:2, II:1, II:3, II:4, II:5, and the proband's father (Figure 6).

### Truncated Protein

**Figure 7:**
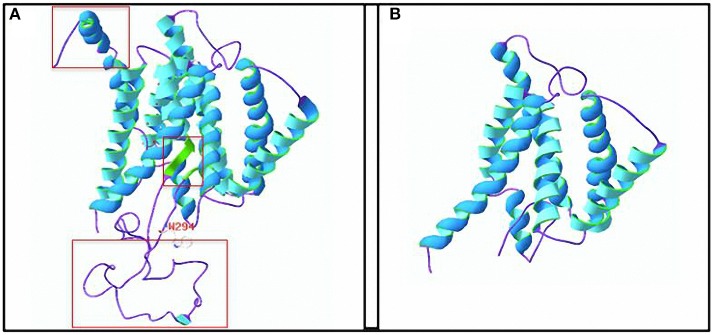
Three-dimensional (3D) structure of the human presenilin 1 protein. **(A)** 3D structure of the wild-type protein. The a-helix is shown in blue, the β-sheet is shown in green, and a random coil is shown in purple. **(B)** 3D structure of the truncated protein. The truncation is at the W294 amino acid. The red box represents the apparent difference in protein structure caused by the truncation. Amino acid residue structure at W294: white, red, and blue represent C, O, and N atoms, respectively.

The SWISS-MODEL server predicted that the amino acid sequence from the mutated *PSEN1* gene had a truncation mutation at amino acid 294 (p.W294^*^), leading to significant differences in the spatial structure of the protein (Figure 7). The template used to predict the 3D structure of the human presenilin 1 protein was PDB ID: 6iyc.1. B, and 99.79% sequence homology was observed. The predicted results indicate that the mutation is likely pathogenic.

## DISCUSSION

During the literature search, no reports describing a c.881G>A mutation in exon 14 of the *PSEN1* gene, which is the first nonsense mutation in the *PSEN1* gene, were found. This variant is also absent from the genome database of the normal population (https://gnomad.broadinstitute.org/gene/ENSG00000080815). The codon for the tryptophan at position 294 of the protein was mutated from UGG to the stop codon UAG, resulting in the formation of a truncated protein (p.W294^*^). According to the clinical symptoms, genetic test results, and the Standards and Guidelines for the Interpretation of Sequence Variants: A Joint Consensus Recommendation of the American College of Medical Genetics and Genomics and the Association for Molecular Pathology (12), this variant is classified as “class II: likely pathogenic.”

Nearly 30 clinical symptoms have been reported to be caused by *PSEN1* gene mutations, including forgetfulness and personality changes (13, 14), balance difficulties (15), progressive gait disturbance (16–18), myoclonus (19, 20), extrapyramidal symptoms (21–23), language impairment (24–26), visual structural disorder (16, 27, 28), visual hallucinations (19), vision loss (29), and others. In this study, all three family members with the *PSEN1* mutation presented at a young age with acute encephalopathy as the prominent symptom, characterized by the acute onset of fever, impaired consciousness or mental abnormality, signs of pyramidal tract damage, and meningeal irritation. All affected individuals had seizures at different stages of the disease. In the auxiliary examinations, increased protein levels and normal or slightly elevated WBC counts were found in the CSF. EEG showed extensive abnormal waves in these three individuals, and brain MRI showed extensive lesions in the white matter and subcortex. These results indicate that the patient's symptoms were due to extensive brain lesions and were different from those caused by other *PSEN1* variants that have been reported to date. Combined with the DNA sequencing results, these data led us to hypothesize that the nonsense mutation in the *PSEN1* gene, chr14: 73673106 c.881G>A, was the causative variant of the disease in this family, but additional cases of this familial disease are needed to support this hypothesis.

The clinical manifestations of this family need to be identified from some diseases. As to acute onset and reversible encephalopathy, we need to exclude some metabolic diseases, such as drug-induced (including the illegal) cases, environmental factors, and posterior reversible encephalopathy syndrome. The proband and his two sisters did not have a history of contact in this regard and related underlying diseases, and they developed fever in the earliest stage of onset. So these diseases can be excluded. The widespread white matter lesion with family history also needs to be distinguished from hereditary leukoencephalopathy. However, the clinical features of these diseases are chronic onset, progressive development, and mostly suffering from childhood (such as metachromatic leukodystrophy, Alexander disease, Pelizaeus–Merzbacher disease, and adrenoleukodystrophy) or stroke-like onset [for example, cerebral autosomal dominant arteriopathy with subcortical infarcts and leukoencephalopathy (CADASIL) and cerebral autosomal recessive arteriopathy with subcortical infarcts and leukoencephalopathy (CARASIL)]. Moreover, the brain MR manifestations in these diseases are different from that in the three siblings (30–35). More importantly, whole-exome sequencing did not detect gene mutations that caused these diseases (including SUMF1, GFAP, PLP1, ABCD1, NOTCH3, HTRA1). So these diseases can also be ruled out. A paper reported that acute-onset encephalopathy was genetically associated with RANBP2 mutation (36).

Retinitis pigmentosa is a hereditary retinopathy characterized by progressive loss of rods and cones and causes severe visual dysfunction and eventual bilateral blindness (37). In this family, the proband and his two sisters began to experience a progressive decline in bilateral vision during adolescence. The fundus photograph in Figure 4 shows obvious retinal pigmentation in patients III:4 and III:6, who had significant reductions in visual acuity. Because the proband's mother and maternal grandfather had progressive bilateral vision loss at a young age, we suspect that the *PSEN1* gene mutation in this family is also associated with hereditary retinal pigmentation and may be a common causative gene for neurological damage and retinitis pigmentosa. Animal and cell research have shown that presenilin regulates retinotectal synapse formation through EphB2 receptor processing (38). However, no reports on *PSEN1* gene mutations in patients with retinitis pigmentosa are available, although the mutations in at least 96 genes have been reported to be responsible for the disease (https://sph.uth.edu/Retnet/).

There are some limitations of this study. Because two family members (II:2 and I:1) were deceased, it was not clear whether they had similar neurological diseases and retinopathy. We also failed to verify whether they had the same gene mutation. This study reveals that the c.881G>A mutation in the *PSEN1* gene is a possible pathogenic mutation, and further studies in cell and animal models harboring this mutation are needed.

## Conclusions

A novel *PSEN1* gene mutation, chr14: 73673106 c.881G>A, may result in acute encephalopathy and may be a causative mutation for retinitis pigmentosa.

## Data Availability Statement

All datasets generated for this study are included in the article/supplementary material.

## Ethics Statement

The studies involving human participants were reviewed and approved by the Ethics Committee of Sun Yat-sen Memorial Hospital, Sun Yat-sen University, China. The patients/participants provided their written informed consent to participate in this study. Written informed consent was obtained from the individual(s) for the publication of any potentially identifiable images or data included in this article.

## Author Contributions

YW designed the study and performed the history-taking and physical examination. CY performed the experiments And wrote the manuscript with WZ. LD and ZL analyzed the data. XG and VZ analyzed gene mutation.

## Conflict of Interest

The authors declare that the research was conducted in the absence of any commercial or financial relationships that could be construed as a potential conflict of interest.
